# Global trends and hotspots of natural medicines for pathological scar treatment: a visualized bibliometric analysis (2008–2025)

**DOI:** 10.3389/fmed.2026.1787448

**Published:** 2026-03-23

**Authors:** Ling Ge, Chuying Li, Xianchun Zhou, Longquan Pi, Zhehu Jin

**Affiliations:** 1 Department of Dermatology, The Affiliated Hospital of Yanbian University (Yanbian Hospital), Jilin, China; 2 Department of Medical Cosmetology, The Affiliated Hospital of Yanbian University (Yanbian Hospital), Jilin, China; 3 Yanbian University Medical College, Jilin, China; 4 Department of Internal Medicine, The Affiliated Hospital of Yanbian University (Yanbian Hospital), Jilin, China

**Keywords:** bibliometric analysis, microneedles, nanomaterials, natural medicines, pathological scar

## Abstract

**Background:**

The therapeutic role of natural medicines in pathological scars has received widespread attention in recent years. However, there is no systematic bibliometric study to analyze the research hotspots and trends in this field.

**Purpose:**

This study aimed to provide researchers with a comprehensive summary of natural medicines in pathological scars; visualize the current research status, hotspot directions, and development trends; and offer references for subsequent research and clinical translation in this field.

**Methods:**

Publications related to the topic from 2008 to 2025 were retrieved from the WOSCC and PubMed databases. Bibliometric analyses were performed using VOSviewer, CiteSpace, Scimago Graphica, Microsoft Office Excel, and GraphPad Prism.

**Results:**

Global publications on natural medicines for pathological scar treatment have steadily increased. A total of 234 papers were retrieved, contributed by 1,395 authors from 396 institutions across 39 countries/regions. China far outnumbered other countries in publication output, with four of the top five authors residing in China. Shanghai Jiao Tong University ranked first among institutions by publication count. The most recent keywords—drug delivery, transdermal delivery, antioxidant, angiogenesis, and the transforming growth factor-β (TGF-β)/Smad—suggest emerging research frontiers.

**Conclusion:**

Research on natural medicines in pathological scars is still in a developmental stage. This is the first bibliometric analysis in this domain, highlighting the current hotspots and shedding light on future research directions.

## 1 Introduction

Pathological scars are fibroproliferative lesions that form during the wound healing process of skin tissue (1). Disruptions in regulatory mechanisms lead to the excessive proliferation and abnormal arrangement of collagen fibers, resulting in lesions that exceed the scope of normal physiological repair and are accompanied by functional impairments or cosmetic abnormalities (2–4). They represent a typical manifestation of abnormal wound healing. Clinically, pathological scars are primarily categorized into two types: hypertrophic scar (HS) and keloid (5). The formation of these scars not only affects the patient’s appearance but may also cause functional disabilities, severely impacting their quality of life. To date, the specific mechanisms underlying pathological scar formation remain unclear. Current treatment methods mainly include surgery, local injections, such as corticosteroids and botulinum toxin type A, laser therapy, radiation therapy, and topical silicone gel (6–10). However, these approaches fail to achieve satisfactory therapeutic outcomes. Therefore, researching and developing more effective anti-scar medications with fewer side effects is of crucial importance.

Natural medicines are widely favored due to their low cost, easy accessibility, safety, and efficacy; nearly 80% of the population in developing countries uses herbal medicines for wound and scar treatment (11–13). In recent years, a large body of research has demonstrated the potential of natural medicines in promoting wound healing and treating pathological scars, and natural medicinal substances such as asiaticoside, epigallocatechin-3-gallate (EGCG), quercetin, onion extract, shikonin, and 20(R)-ginsenoside Rg3 have been proven to exhibit therapeutic effects in anti-fibrosis, antioxidant, and anti-inflammatory aspects (14–19). However, the rapid surge in publications within this field may hinder researchers from fully grasping the key progress and future directions of natural medicines applied to pathological scar treatment. Thus, a systematic analysis of the research hotspots and trends in this specific area is essential. Bibliometrics, as an emerging knowledge integration approach, enables the identification of both quantitative and qualitative characteristics of publications while exploring prominent research trends in a particular field. To date, no bibliometric studies have been carried out on the interdisciplinary area of pathological scars and natural medicines. This research intended to conduct a bibliometric analysis of publications centered on natural medicines in pathological scar studies—encompassing aspects such as publication quantity, leading contributing countries, institutions, journals, and keywords. It aimed to summarize current research hotspots and pinpoint existing problems, and the findings of this study are anticipated to offer guidance for researchers dedicated to investigating natural medicines for pathological scar treatment.

## 2 Materials and methods

### 2.1 Data acquisition and search strategy

**Figure 1:**
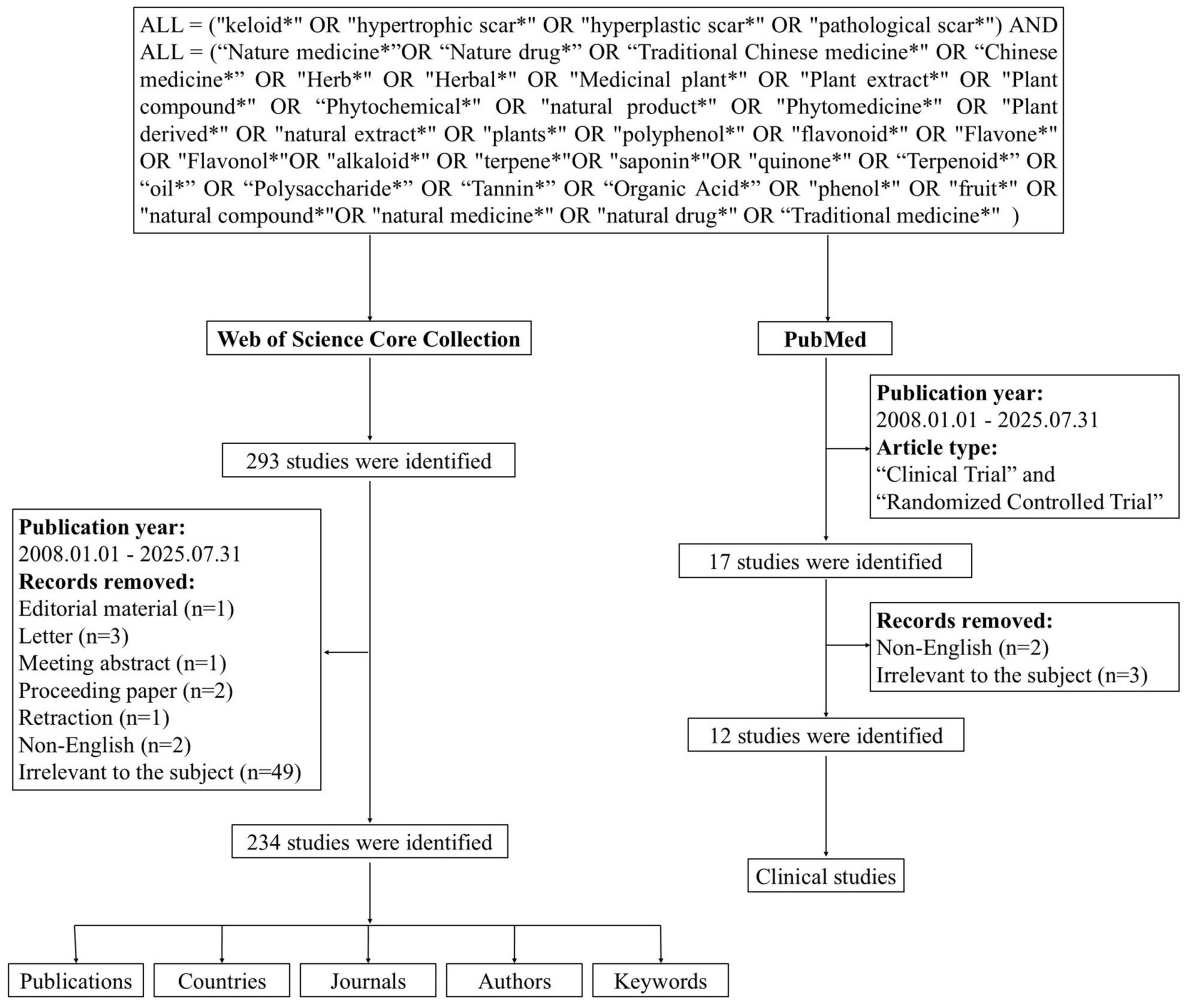
Flow diagram for bibliometric screening in this study. Flow diagram showing the two independent literature retrieval and screening processes for the core bibliometric corpus (WoSCC) and clinical supplementary analysis corpus (PubMed). Irrelevant studies were excluded by abstract review, with 234 articles and 12 clinical trials finally included for subsequent analysis, respectively.

The Web of Science Core Collection (WoSCC) is the core and foundational database on the WoS platform. We used this database for a comprehensive visual analysis of publications, contributing countries, journals, authors, and keywords, whereas PubMed^1^ was exclusively used for descriptive analysis of clinical study characteristics. A systematic literature search was conducted on 1 August 2025. For the WoSCC, the search period was set from January 2008 (the year the first relevant study was published) to July 2025; document types were restricted to articles and review articles, with non-English literature excluded. A preliminary search identified 293 documents, of which 49 irrelevant to the research topic were excluded following abstract screening, resulting in the final inclusion of 234 documents. For PubMed, the search topic and time range were consistent with those for the WoSCC, while document types were limited to clinical trials and randomized controlled trials. A total of 17 relevant documents were initially retrieved from PubMed; 2 non-English publications and 3 irrelevant studies were subsequently excluded, with 12 articles finally included in the analysis (Figure 1).

### 2.2 Bibliometric analysis

Data analysis and management were conducted using VOSviewer 1.6.20, CiteSpace 6.4. R1, Scimago Graphica 1.0.51, Microsoft Office Excel 2021, and GraphPad Prism 10.1.2. VOSviewer 1.6.20 was used for collaborative network analysis of countries/regions, institutions, and authors; co-citation analysis of references, journals, and authors; and keyword co-occurrence analysis. For collaborative network analysis, the minimum occurrence frequencies for countries/regions, institutions, and authors were set at 1, 1, and 2, respectively. In co-citation analysis, the minimum occurrence frequencies for references, journals, and authors were set at 5, 20, and 10, respectively. Scimago Graphica 1.0.51 was used to analyze country/region collaboration. CiteSpace 6.4. R1 was used for keyword clustering analysis, timeline visualization, and burst detection. The time range was set from January 2008 to July 2025 with a 1-year time slice; in the network pruning settings, “pruning sliced networks” was selected, other settings remained default, and the analysis was performed based on keywords (20). Microsoft Office Excel 2021 was used for data management, statistical analysis of the distribution of literature types and disciplines, and creation of relevant statistical charts. GraphPad Prism 10.1.2 was used to generate annual publication and citation statistics charts.

## 3 Results

### 3.1 Basic trend analysis

**Figure 2:**
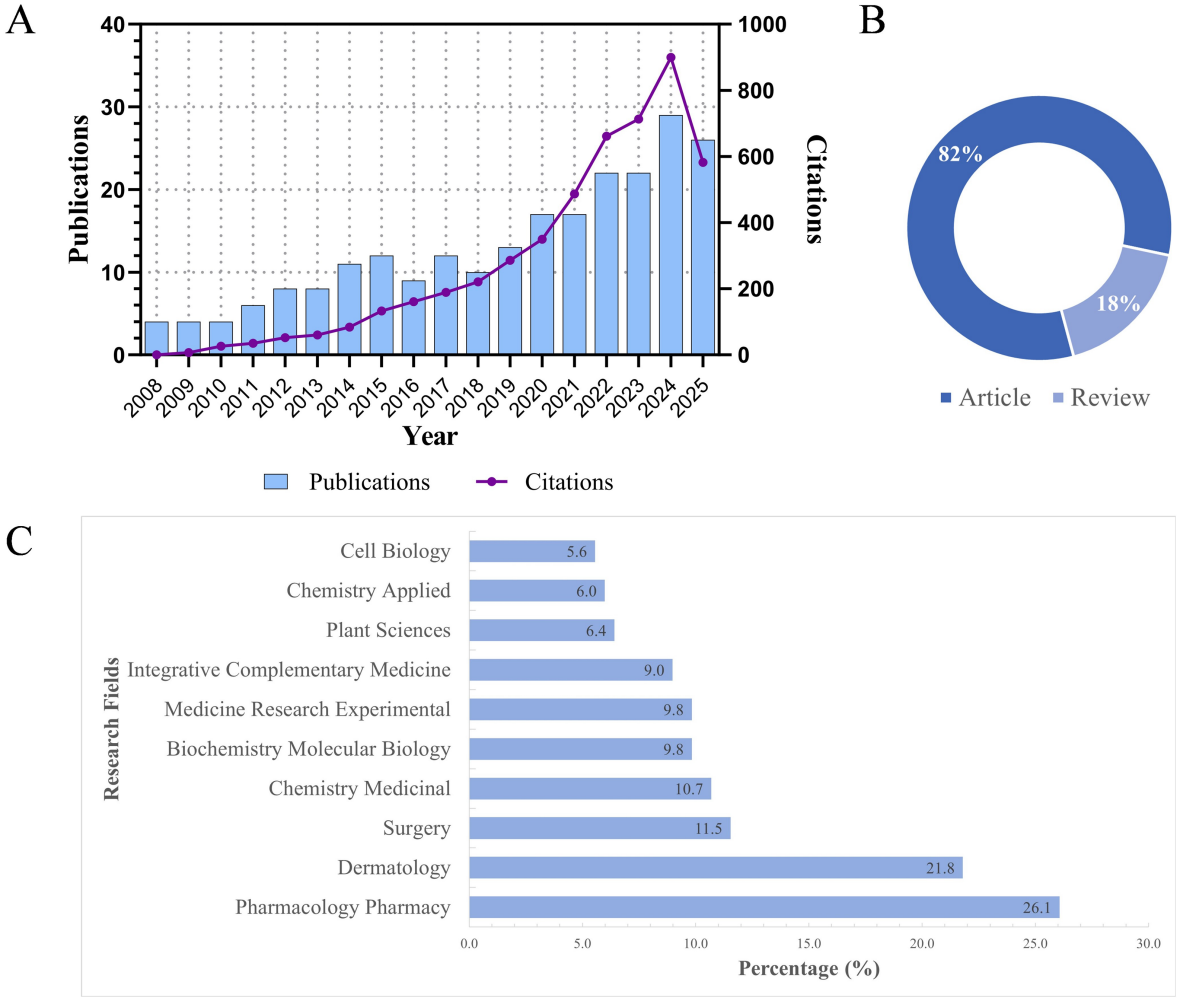
Basic overview of the retrieved articles on natural medicines for pathological scar treatment. **(A)** Annual publications and normalized citation data from January 2008 to July 2025, reflecting the overall research output and citation influence trend; **(B)** distribution of document types (original articles and review articles) in the included core corpus; and **(C)** top 10 disciplinary classifications of the included studies with corresponding percentage proportions.

The results indicated that this study retrieved literature related to natural medicines and pathological scars from the WoSCC database, covering the period from January 2008 to July 2025. After excluding papers irrelevant to the research topic, a total of 234 documents were obtained, including 193 research articles and 41 review articles. As presented in Figure 2, since 2008, the number of publications has exhibited an overall upward trend, reaching a relatively high level around 2024. The number of citations has also shown an increasing pattern, with a significant growth rate. According to statistics from Web of Science categories, the top three categories are pharmacology and pharmacy (61 articles, 26.1%), dermatology (51 articles, 21.8%), and surgery (27 articles, 11.5%).

### 3.2 Collaboration network analysis

#### 3.2.1 Countries/regions

**Figure 3:**
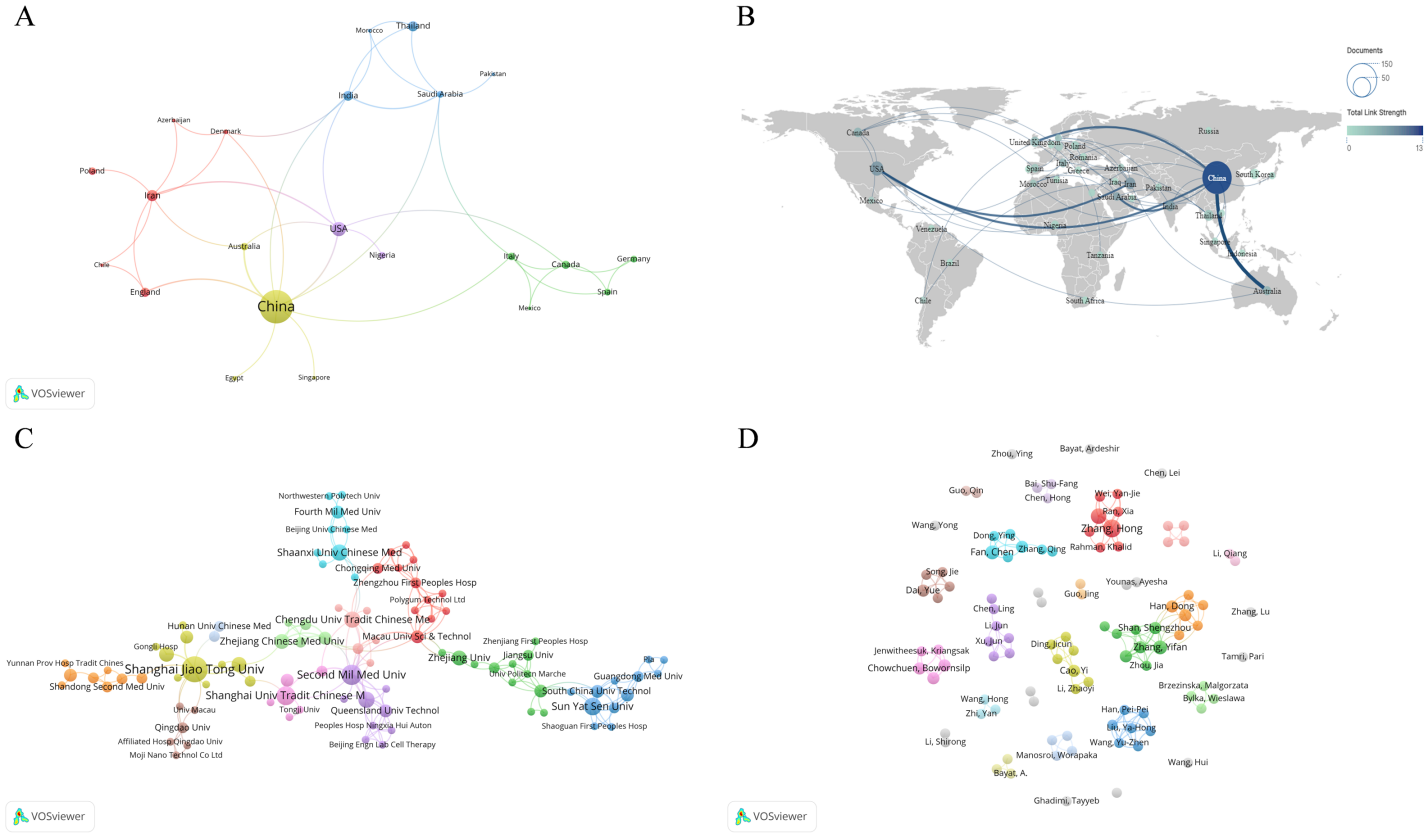
Co-authorship knowledge maps. **(A,B)** Country/regional co-authorship network (node size = publication quantity, line thickness = collaborative connection strength, different colors = clustering groups); **(C)** Institutional co-authorship network (top productive institutions are visualized with collaborative links); and **(D)** Author co-authorship network (node size = author’s publication quantity, line thickness = inter-author collaboration strength).

**Figure 4:**
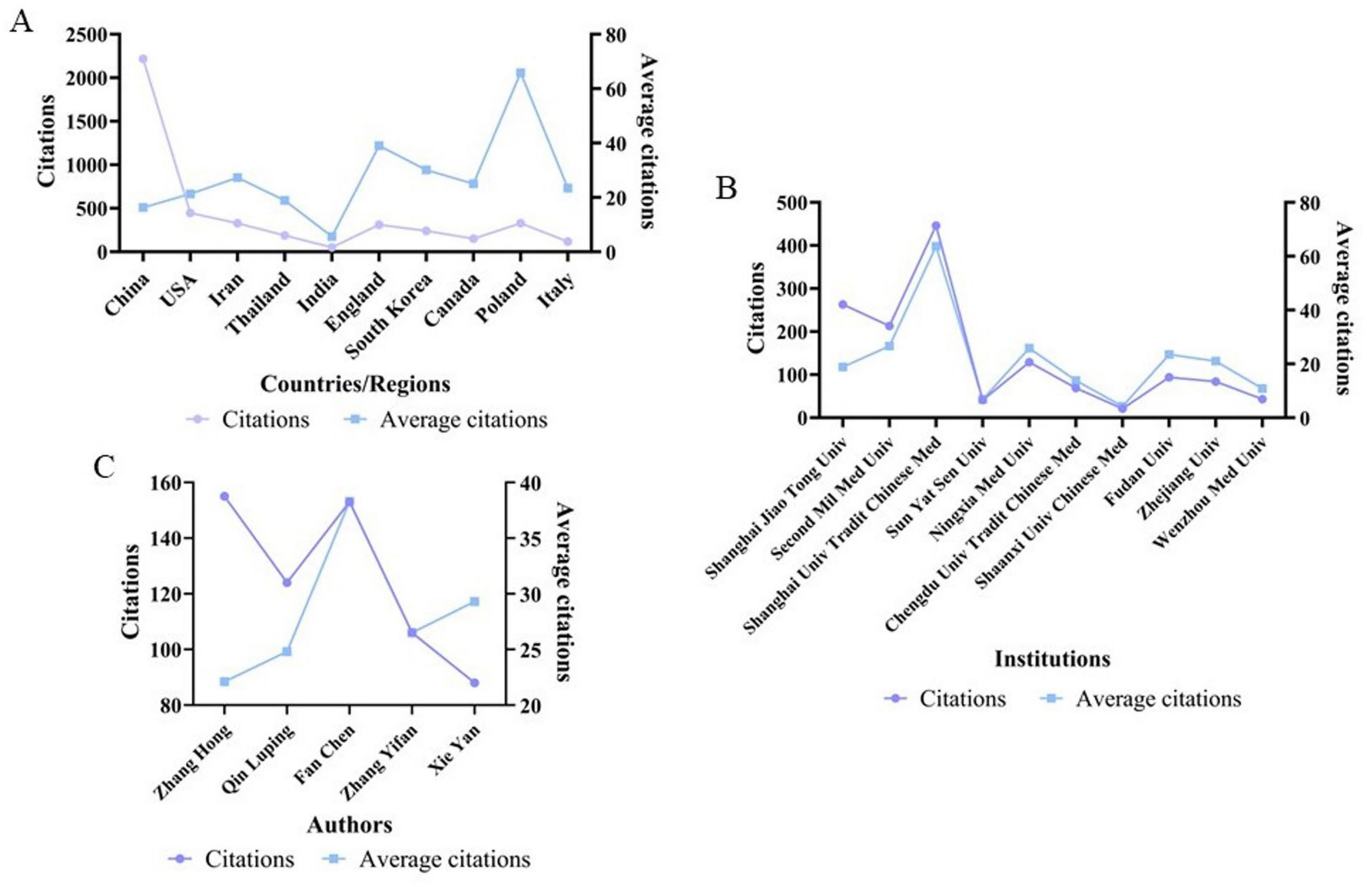
Citation analysis of the top contributing countries/regions, institutions, and authors. **(A)** Citations and ACs of the top 10 productive countries/regions in this field, reflecting the academic influence of different countries/regions; **(B)** citations and ACs of the top 10 research institutions, showing the citation performance of core research institutions; and **(C)** citations and ACs of key contributing authors, indicating the academic influence of core researchers in this field.

Approximately 39 countries and regions worldwide have conducted research on the application of natural medicines in pathological scar studies, with publications appearing in 149 journals. As shown in Table 1, the three most active countries in this field are China (136 articles, 16.3 average citations [ACs]), the United States (USA) (21 articles, 21.3 ACs), and Iran (12 articles, 27.3 ACs). Among the top 10 countries in terms of publication quantity, Poland, which ranks ninth, has the highest AC (65.8) by a significant margin. This indicates that although Poland has published fewer articles, its research exhibits high quality and strong influence. Overall, the total link strength (TLS) among all countries is not high, and China ranks first with a TLS of 13, having collaborative relationships with the USA, Australia, and England (Figures 3A,B, 4A).

**Table 1:** Top 10 most active countries/regions, institutions, and authors.

Item	Ranking based on the number of published articles	Name	Documents	Citations	Average citations	Ranking based on link strength with other countries	Name	Total link strength
Countries/Regions	1	China	136	2,217	16.3	1	China	13
	2	USA	21	447	21.3	2	Iran	8
	3	Iran	12	328	27.3	3	USA	8
	4	Thailand	10	189	18.9	4	India	7
	5	India	9	50	5.6	5	Saudi Arabia	7
	6	England	8	312	39	6	Australia	5
	7	South Korea	8	241	30.1	7	Canada	5
	8	Canada	6	150	25	8	Italy	5
	9	Poland	5	329	65.8	9	Denmark	4
	10	Italy	5	117	23.4	10	England	4
Institutions	1	Shanghai Jiao Tong University	14	263	18.8	1	Second Military Medical University	19
	2	Second Military Medical University	8	213	26.6	2	Ningxia Medical University	16
	3	Shanghai University of Traditional Chinese Medicine	7	446	63.7	3	Shanghai Jiao Tong University	15
	4	Sun Yat Sen University	6	41	6.8	4	Sun Yat Sen University	12
	5	Ningxia Medical University	5	129	25.8	5	Macau University of Science and Technology	12
	6	Chengdu University of Traditional Chinese Medicine	5	69	13.8	6	South China University of Technology	12
	7	Shaanxi University of Chinese Medicine	5	21	4.2	7	Chengdu University of Traditional Chinese Medicine	11
	8	Fudan University	4	94	23.5	8	Shanghai Seventh People’s Hospital	11
	9	Zhejiang University	4	84	21	9	Zhengzhou First People’s Hospital	11
	10	Wenzhou Medical University	4	43	10.8	10	Queensland University of Technology	10
Authors	1	Zhang, Hong	7	155	22.1	1	Zhang, Yifan	18
	2	Qin, Lu-Ping	5	124	24.8	2	Zhang, Hong	17
	3	Fan, Chen	4	153	38.3	3	Qin, Lu-Ping	15
	4	Zhang, Yifan	4	106	26.5	4	Shan, Shengzhou	15
	5	Xie, Yan	3	88	29.3	5	Li, Qingfeng	13

#### 3.2.2 Institutions

A total of 396 institutions have contributed to research on natural medicines and pathological scars. All top 10 institutions in terms of publication quantity are from China (Table 1; Figures 3C, 4B), among which Shanghai Jiao Tong University ranks first with 14 publications (18.8 ACs). It is followed by the Second Military Medical University (8 articles, 26.6 ACs) and the Shanghai University of Traditional Chinese Medicine (7 articles, 63.7 ACs). The Second Military Medical University has the largest collaboration network (with a TLS of 19), while the Shanghai University of Traditional Chinese Medicine ranks first in ACs.

#### 3.2.3 Authors

Nearly 1,400 researchers are engaged in studies on natural medicines and pathological scars (Table 1; Figures 3D, 4C). Zhang Hong from the Second Military Medical University is the most productive author, with 7 published articles (22.1 ACs), followed by Qin Luping from the Second Military Medical University (5 articles, 24.8 ACs) and Fan Chen from the Queensland University of Technology (4 articles, 38.3 ACs). However, notably, Zhang Yifan, Zhang Hong, and Qin Luping have closer collaborative relationships with other authors.

### 3.3 Journal analysis

**Figure 5:**
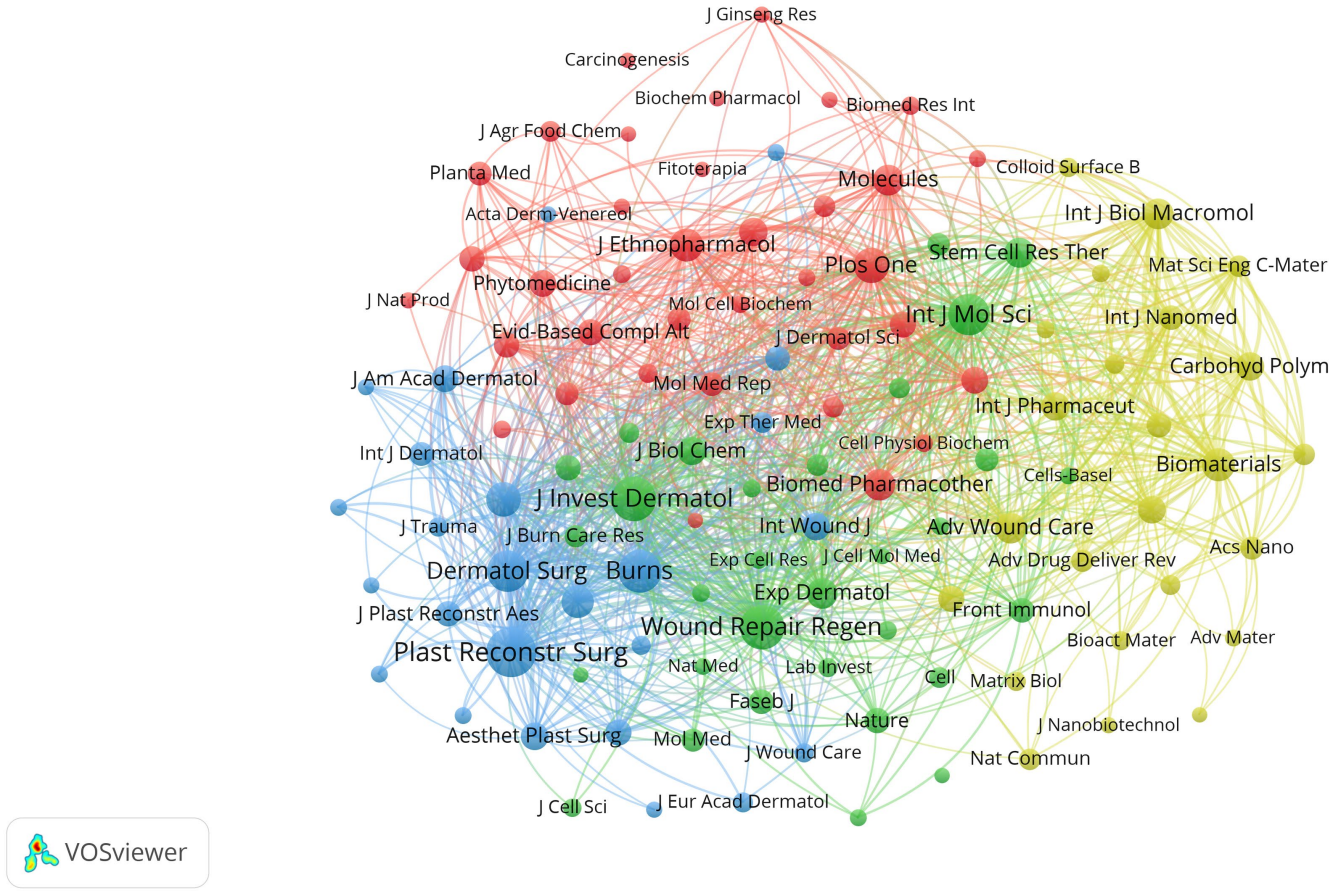
Journal co-citation knowledge map. Visualization of the journal co-citation network in this research field, where node size represents journal co-citation frequency, line thickness represents the co-citation strength between journals, and different colors represent clustered journal groups with close academic correlations.

As of July 2025, 149 science citation index (SCI) journals have published articles related to natural medicines and pathological scars. Table 2 summarizes the top 10 journals by citation count, as analyzed by VOSviewer. Among these, *Experimental and Therapeutic Medicine* has published 3 articles and garnered the highest number of citations (295), followed by the *International Journal of Biological Macromolecules* (286 citations) and the *Journal of Tissue Viability* (201 citations). The *Journal of Tissue Viability*, *American Family Physician*, and *Postepy Dermatologii i Alergologii* rank among the top three in terms of average citations per article. Six of the ten journals belong to the Journal Citation Reports (JCR) Q1 category, indicating high academic influence. The USA is the country with the largest share of publications among the top 10 journals. As shown in Figure 5, *Plastic and Reconstructive Surgery* ranks first among co-cited journals with 267 co-citations.

**Table 2:** Top 10 most cited journals.

Rank	Journal	Documents	Total citations	Average citations	IF (2024)	JCR	Country
1	*Experimental and Therapeutic Medicine*	3	295	98.3	2.3	Q3	Greece
2	*International Journal of Biological Macromolecules*	4	286	71.5	8.5	Q1	Netherlands
3	*Journal of Tissue Viability*	1	201	201.0	2.8	Q2	England
4	*Journal of Investigative Dermatology*	3	192	64.0	5.7	Q1	USA
5	*Phytotherapy Research*	2	187	93.5	6.3	Q1	England
6	*American Family Physician*	1	178	178.0	3.5	Q1	USA
7	*Archives of Dermatological Research*	6	163	27.2	2.1	Q3	Germany
8	*Aesthetic Plastic Surgery*	4	133	33.3	2.8	Q1	USA
9	*Postepy Dermatologii i Alergologii*	1	131	131.0	1.7	Q3	Poland
10	*Pharmaceutical Biology*	4	124	31.0	4.8	Q1	Netherlands

### 3.4 Literature analysis

From the perspective of literature analysis, the references with the highest citation counts are shown in Table 3. The article with the highest number of citations was published by Ren et al. (21) in *Experimental and Therapeutic Medicine* in 2019, with 283 citations. This article reviews the therapeutic applications and potential mechanisms of kaempferol—a natural flavonoid compound found in tea, fruits, and vegetables—in various diseases, including acute and chronic inflammatory disorders (e.g., colitis), cancers (e.g., esophageal cancer, breast cancer), and fibroproliferative diseases (e.g., HS). The second most cited article was published by Gaspar-Pintiliescu et al. (22) in the *International Journal of Biological Macromolecules* in 2019, with 246 citations. It focuses on exploring the application advantages and effects of natural composite dressings formed by combining collagen/gelatin with plant bioactive compounds in wound healing while also pointing out existing problems and providing prospects for future research directions. The third most cited article was published by Oryan et al. (23) in the *Journal of Tissue Viability*, with 201 citations. It systematically elaborates on the biological properties of honey (e.g., antibacterial, anti-inflammatory, and antioxidant effects) and its mechanism of action in wound healing, confirming that honey is a safe, economical, and effective option for wound management, as it can eliminate microbial contamination, promote wound re-epithelialization, and reduce scar formation.

**Table 3:** Top 10 most cited articles.

Rank	Title	Journal	Citations		Publication year	First author
			Total	Average per year		
1	Recent progress regarding kaempferol for the treatment of various diseases	*Experimental and Therapeutic Medicine*	283	40.4	2019	Ren (21)
2	Natural composite dressings based on collagen, gelatin, and plant bioactive compounds for wound healing: A review	*International Journal of Biological Macromolecules*	246	35.1	2019	Gaspar-Pintiliescu (22)
3	Biological properties and therapeutic activities of honey in wound healing: A narrative review and meta-analysis	*Journal of Tissue Viability*	201	20.1	2016	Oryan (23)
4	Management of Keloids and Hypertrophic Scars	*American Family Physician*	178	10.5	2009	Juckett (43)
5	*Centella asiatica* in Dermatology: An Overview	*Phytotherapy Research*	152	12.7	2014	Bylka (44)
6	*Centella asiatica* in cosmetology	*Postepy Dermatologii i Alergologii*	131	10.1	2013	Bylka (45)
7	Green tea polyphenol epigallocatechin-3-gallate suppresses collagen production and proliferation in keloid fibroblasts via inhibition of the STAT3-signaling pathway	*Journal of Investigative Dermatology*	101	5.6	2008	Park (46)
8	Treatment of Hypertrophic Scars With Intralesional Botulinum Toxin Type A Injections: A Preliminary Report	*Aesthetic Plastic Surgery*	95	5.6	2009	Xiao (47)
9	Asiaticoside enhances normal human skin cell migration, attachment, and growth *in vitro* wound healing model	*Phytomedicine*	93	6.6	2012	Lee (48)
10	Combination of argan oil and phospholipids for the development of an effective liposome-like formulation able to improve skin hydration and allantoin dermal delivery	*International Journal of Pharmaceutics*	92	9.2	2016	Manca (49)

**Figure 6:**
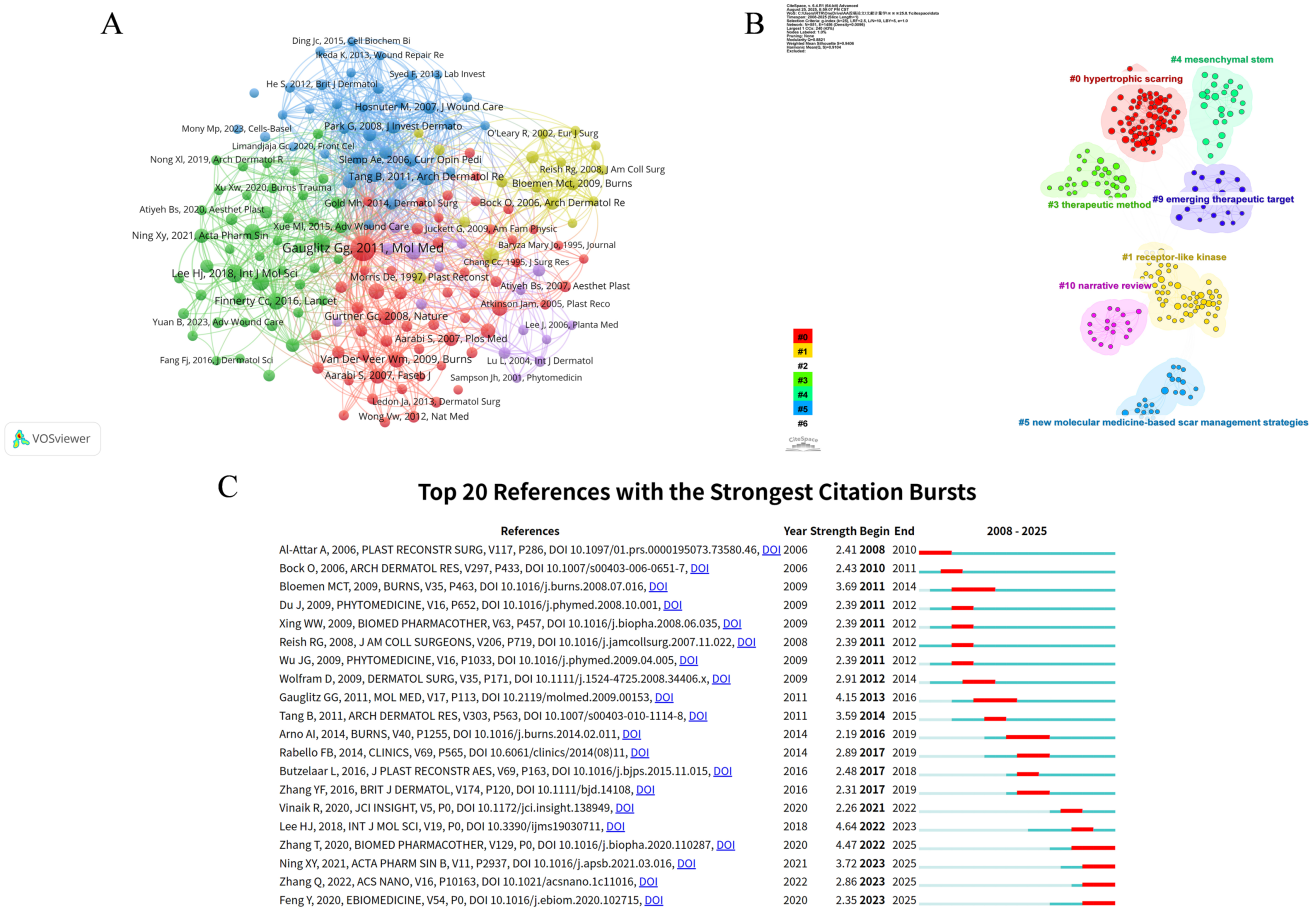
Reference co-citation knowledge maps. **(A)** Network mode of reference co-citation (node size = reference co-citation frequency, line thickness = inter-reference co-citation strength); **(B)** keyword clustering of co-cited references, with different colors representing distinct research theme clusters; and **(C)** top 20 citation burst references, reflecting the references with rapidly increasing citation frequency in specific time periods.

Co-citation refers to the situation where two publications are cited simultaneously by other articles, reflecting the strength of their academic relationship and enabling the identification of the core components in the field. In this study, 234 articles and their 10,756 references were retrieved, and the visualization is shown in Figure 6. Table 4 lists the top 10 co-cited references. All top 10 co-cited references were published more than 5 years ago and in high-quality journals. Seven clusters were identified using CiteSpace software (Figure 6B) (modularity Q = 0.88, silhouette S = 0.94), among which the largest and most recent cluster is “hypertrophic scarring” (Cluster #0). Citation bursts in references refer to a significant increase in the citation frequency of these references over time compared to their usual rate; such studies can help explore how research hotspots have evolved over the years. The reference with the strongest burst strength (strength = 4.64, burst period = 2022–2023) is a review published in the International Journal of Molecular Sciences in 2018, which focuses on the biology, prevention, and treatment strategies of HS and keloid (24). Currently, there are still four articles widely cited whose main content involves research on the transforming growth factor-β/Smad (TGF-β/Smad) signaling pathway, drug delivery, and the application of microneedles in the treatment of pathological scars (Figure 6C) (25–28).

**Table 4:** Top 10 most cited references.

Rank	Title	Journal	JCR	Citations	Publication year	First author
1	Hypertrophic Scarring and Keloids: Pathomechanisms and Current and Emerging Treatment Strategies	*Molecular Medicine*	Q1	44	2011	Gauglitz (50)
2	Recent Understandings of Biology, Prophylaxis, and Treatment Strategies for Hypertrophic Scars and Keloids	*International Journal of Molecular Sciences*	Q1	20	2018	Lee (24)
3	Wound repair and regeneration	*Nature*	Q1	18	2008	Gurtner (51)
4	Asiaticoside suppresses collagen expression and TGF-β/Smad signaling through inducing Smad7 and inhibiting TGF-βRI and TGF-βRII in keloid fibroblasts	*Archives of Dermatological Research*	Q3	18	2011	Tang (52)
5	Current potential therapeutic strategies targeting the TGF-β/Smad signaling pathway to attenuate keloid and hypertrophic scar formation	*Biomedicine & Pharmacotherapy*	Q1	18	2020	Zhang (25)
6	Hypertrophic scarring: the greatest unmet challenge after burn injury	*Lancet*	Q1	17	2016	Finnerty (53)
7	Keloids and Hypertrophic Scars: Pathophysiology, Classification, and Treatment	*Dermatologic Surgery*	Q2	16	2017	Berman (54)
8	Keloid and Hypertrophic Scars Are the Result of Chronic Inflammation in the Reticular Dermis	*International Journal of Molecular Sciences*	Q1	16	2017	Ogawa (55)
9	Potential cellular and molecular causes of hypertrophic scar formation	*Burns*	Q2	16	2009	Van der Veer (56)
10	On the nature of hypertrophic scars and keloids: a review	*Plastic And Reconstructive Surgery*	Q1	15	1999	Niessen (57)

### 3.5 Keyword analysis

**Figure 7:**
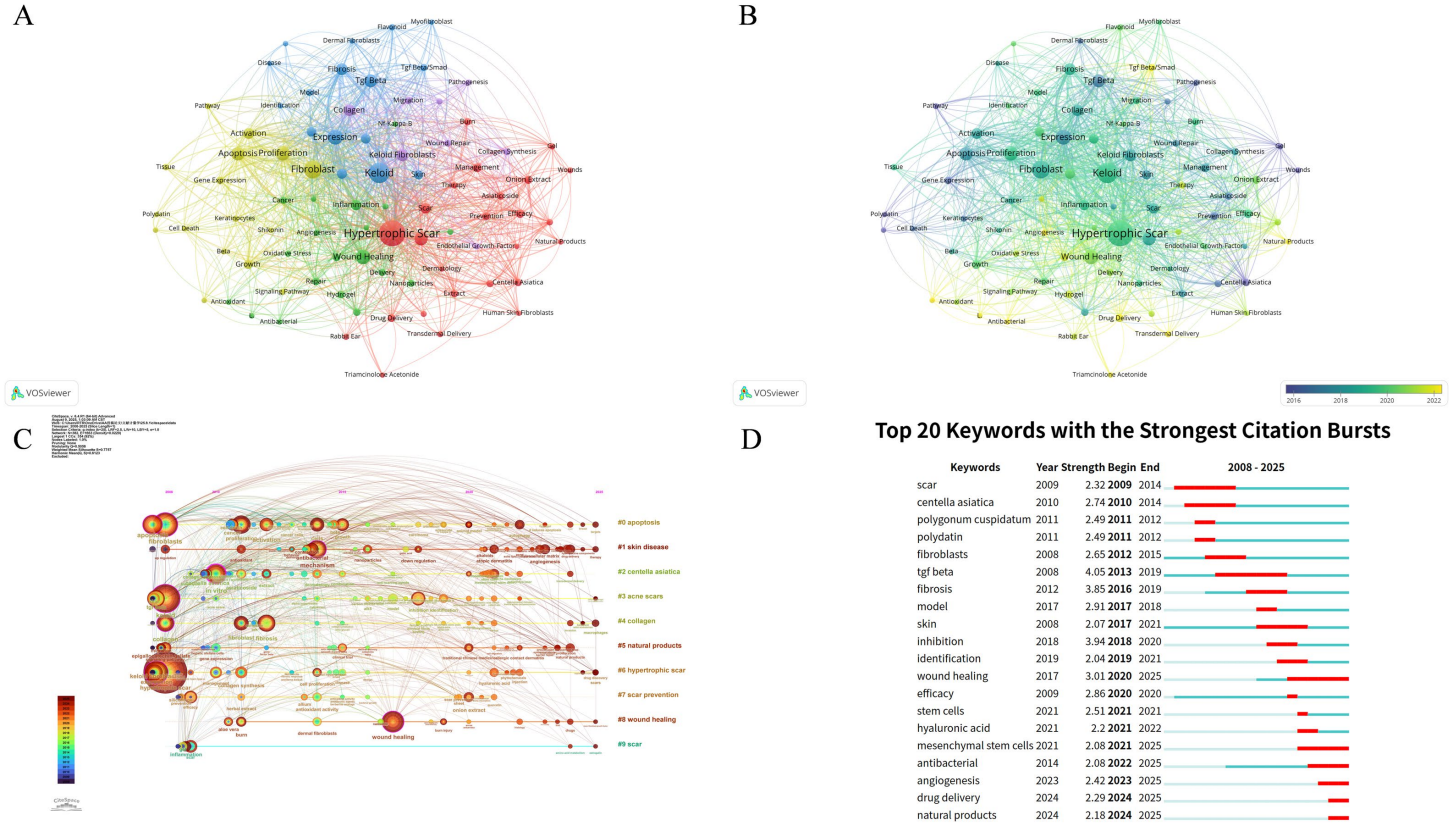
Keyword co-occurrence maps. **(A)** Network mode of keyword co-occurrence (node size = keyword frequency, line thickness = co-occurrence strength, different colors = research theme clusters); **(B)** Overlay mode [color gradient represents the average occurrence year of keywords, from dark blue (early) to yellow (recent)]; **(C)** Timeline of keyword evolution, showing the temporal distribution of core research keywords; and **(D)** Top 20 keywords with the strongest citation bursts, indicating emerging and hot research keywords in different periods.

Co-occurrence analysis of keywords enables a rapid and clear understanding of a research field and its key directions. Among the 1,224 keywords extracted from the 234 articles, the high-frequency keywords (excluding search terms and keywords with little practical significance) identified through the VOSviewer-based co-occurrence analysis (Figure 7A) include wound healing, proliferation, apoptosis, *in vitro*, TGF-β, inflammation, mechanism, and drug delivery. These keywords can be roughly categorized into three clusters: therapy and management of pathological scars using natural medicines (red cluster): this cluster includes keywords such as “Therapy,” “Management,” “Asiaticoside,” “*Centella Asiatica*,” “Onion Extract,” and “Drug Delivery”; pathological mechanisms of pathological scars (blue and purple clusters): keywords in this cluster include “Keloid,” “Hypertrophic Scar,” “Fibrosis,” “Collagen,” “TGF-β,” “Pathogenesis,” “Migration,” and “Identification”; application of natural medicines in wound healing and scar prevention (yellow and green clusters): this cluster covers keywords such as “Wound Healing,” “Wound Repair,” “Tissue,” “Inflammation,” “Gene Expression,” “Antioxidant,” “Oxidative Stress,” and “Apoptosis.” As shown in Figure 7B, keywords are color-coded based on their average year of occurrence: the earliest appearing keywords are shown in dark blue, while the most recent ones are in yellow. The latest keywords in this field include “drug delivery,” “transdermal delivery,” “antioxidant,” “angiogenesis,” “TGF-β/Smad,” and “natural medicines,” reflecting the latest research focuses. Figures 7C,D (density = 0.02, modularity Q = 0.51, and silhouette S = 0.78) present the timeline and burst detection of keywords related to natural medicines and pathological scars since 2008. Keywords that have maintained a continuous burst state—such as “wound healing,” “angiogenesis,” “drug delivery,” and “natural medicines”—indicate potential research hotspots in the future.

### 3.6 Clinical studies

**Figure 8:**
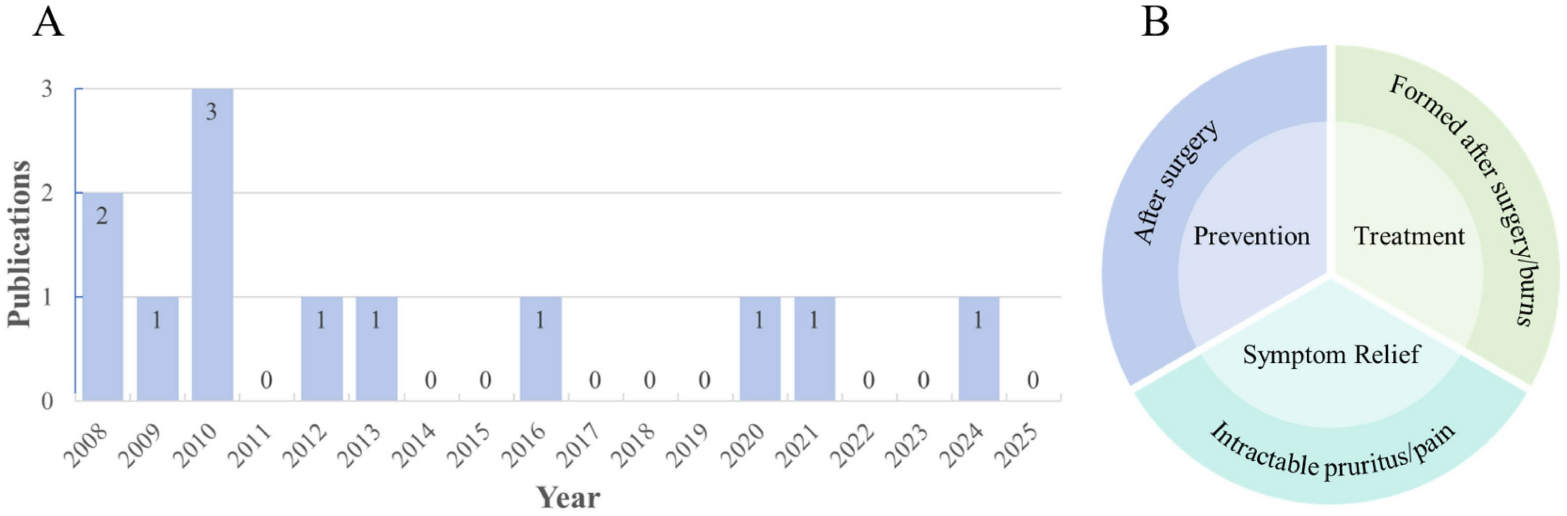
Clinical studies. **(A)** Annual publication distribution of 12 included clinical trials from January 2008 to July 2025, reflecting the temporal publication trend of clinical research in this field, and **(B)** classification of research types of clinical trials, including prevention of pathological scar formation, inhibition of scar progression, and symptomatic relief.

To further elucidate the current clinical status of natural medicines in the treatment of pathological scars, a literature search with the same theme and time scope was conducted in the PubMed database. A total of 12 published clinical studies on natural medicines for pathological scars were retrieved (Figure 8A). The years 2010, 2021, and 2024 were relatively concentrated publication nodes for the studies. The core drugs are represented by onion extract, asiatic acid, and peppermint oil, while the dosage forms are predominantly topical preparations such as gels, ointments, and lotions (Table 5). The study designs were mainly randomized controlled trials and prospective controlled studies, with some including open-label trials. It can be categorized into three main types: prevention of pathological scar formation, inhibition of pathological scar progression, and treatment of associated symptoms (Figure 8B). They focus on the research hotspot of natural medicine screening and optimization of delivery forms.

**Table 5:** Clinical study of natural medicines in the management of hypertrophic scar and keloid.

Category	Natural medicine	Delivery form and administration route	Primary outcomes	Side effects	Disease	Title	Journal	Publication year	First author
Single Natural Medicine Treatment	Mugwort	Lotion (topical application)	78.6% reduction in scar pruritus	—	Hypertrophic scar	Effectiveness of mugwort lotion for the treatment of post-burn hypertrophic scars	*Journal of Plastic Reconstructive and Aesthetic Surgery*	2007	Ogawa (58)
	Onion extract	Gel (topical application)	Marked reduction in lesion volume, pigmentation, and tenderness	Mild acneiform-like eruption, which resolved after drug discontinuation	Hypertrophic scar and keloid	A comparative study evaluating the tolerability and efficacy of two topical therapies for the treatment of keloids and hypertrophic scars	*Journal of Drugs in Dermatology*	2010	Perez (59)
	*Hypericum perforatum*	Ointment (topical application)	Accelerated wound healing and attenuated lesion pain and pruritus	Local irritation, which resolved spontaneously without intervention	Hypertrophic scar	The effect of *Hypericum perforatum* on the wound healing and scar of cesarean	*Journal of Alternative and Complementary Medicine*	2010	Samadi (60)
	Asiatic acid	Gel (topical application)	Suppressed inflammation, increased net skin elasticity, and decreased melanin index	—	Hypertrophic scar	Asiatic acid-entrapped transfersomes for the treatment of hypertrophic scars: *In vitro* appraisal, bioactivity evaluation, and clinical study	*International Journal of Pharmaceutics*	2023	Opatha (61)
Combination of Multiple Natural Medicines	Peppermint oil and menthol	Hydrogel (topical application)	Alleviation of severe, refractory scar pruritus	Skin irritation, which resolved after removal of the hydrogel	Pruritus induced by hypertrophic scar	Effective symptomatic treatment for severe and intractable pruritus associated with severe burn-induced hypertrophic scars: A prospective, multicenter, controlled trial	*Burns*	2016	Wu (62)
	Onion extract and *Aloe vera*	Silicone gel (topical application)	Amelioration of lesion induration, pain, and pruritus	—	Hypertrophic scar and keloid	A comparison of the efficacy of silicone gel containing onion extract and *Aloe vera* to silicone gel sheets to prevent postoperative hypertrophic scars and keloids	*Journal of Cosmetic Dermatology*	2021	Pangkanon (63)
Natural Medicine Combined with Other Agents	Onion extract and triamcinolone acetonide	Gel (topical application) and intralesional injection	Favorable attenuation of pain, pruritus, and scar thickness	—	Hypertrophic scar and keloid	An open, randomized, controlled, comparative study of the combined effect of intralesional triamcinolone acetonide and onion extract gel and intralesional triamcinolone acetonide alone in the treatment of hypertrophic scars and keloids	*Dermatologic Surgery*	2008	Koc (64)

Onion extract and silicone derivative	Gel (topical application)	Improvement in pain, pruritus, and pigmentation	—	Hypertrophic scar	Role of silicone derivative plus onion extract gel in presternal hypertrophic scar protection: a prospective, randomized, double-blinded, controlled trial	*International Wound Journal*	2012	Jenwitheesuk (65)

*Allium cepa*, allantoin, and pentaglycan	Gel (topical application)	Diminished erythema and reduced neovascularization	—	Hypertrophic scar and keloid	Effect of *allium cepa*-allantoin-pentaglycan gel on skin hypertrophic scars: clinical and video-capillaroscopic results of an open-label, controlled, non-randomized clinical trial	*Dermatologic Surgery*	2010	Campanati (20)

*Allium cepa* extract, allantoin and heparin	Gel (topical application)	Significant reductions in scores for pigmentation, vascularity, and scar elevation	—	Hypertrophic scar	The efficacy of onion extract in the management of subsequent abdominal hypertrophic scar formation	*Journal of Wound Care*	2020	Gungor (66)

## 4 Discussion

**Figure 9:**
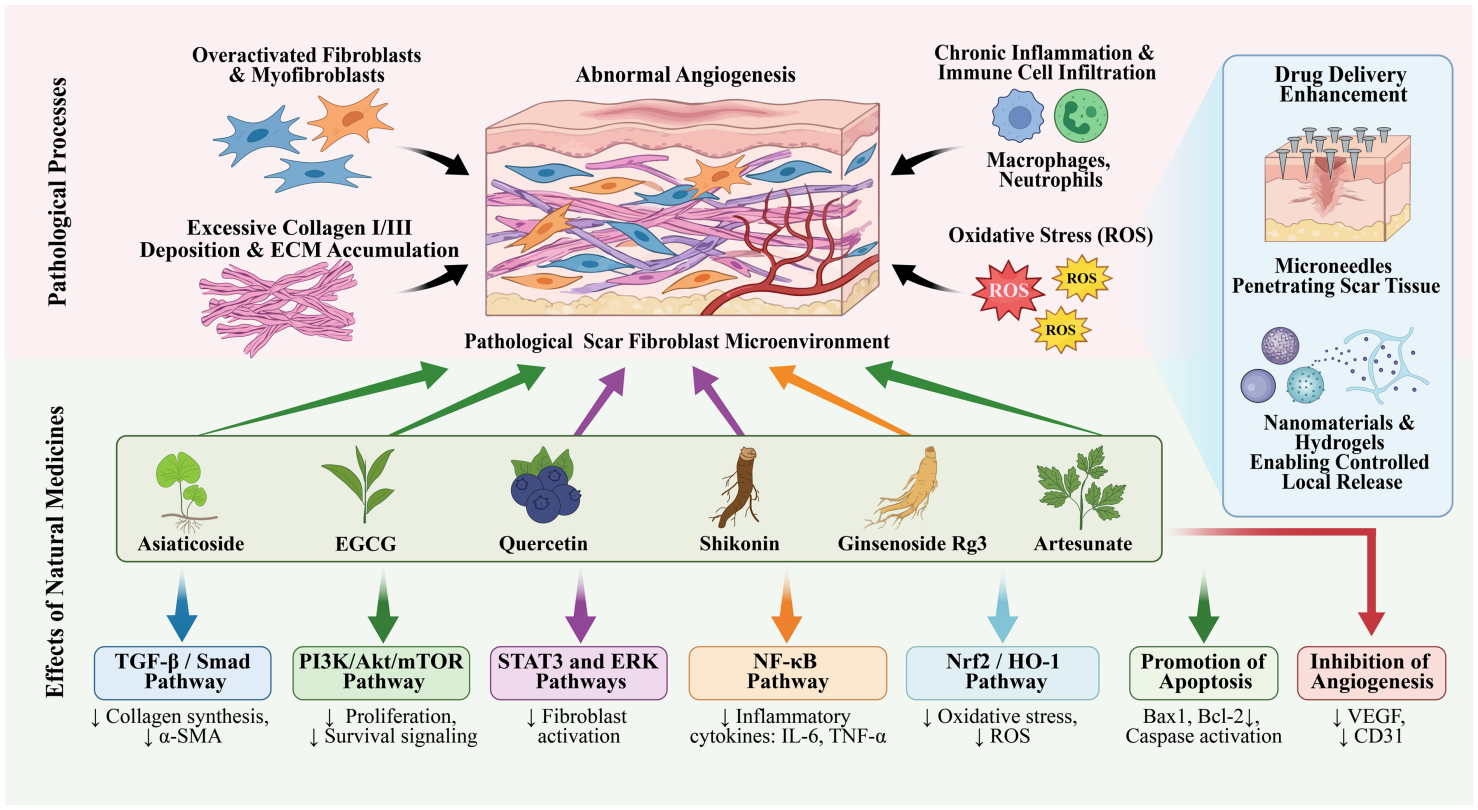
Pathological scars are characterized by fibroblast/myofibroblast overactivation, chronic inflammatory cell (macrophages and neutrophils) infiltration, abnormal angiogenesis, reactive oxygen species (ROS)-mediated oxidative stress, and excessive collagen I/III and excessive extracellular matrix (ECM) deposition. Potent natural medicines (asiaticoside, EGCG, quercetin, shikonin, ginsenoside Rg3, and artesunate) exert therapeutic effects by targeting the above pathological processes. To overcome skin barrier limitations, microneedles (scar tissue penetration), nanomaterials, and hydrogels enable controlled local release of natural medicines, enhancing dermal retention. At the molecular level, these agents regulate multiple pathways: inhibiting TGF-β/mothers against decapentaplegic homologs (Smad), phosphoinositide 3-kinase PI3K/Protein kinase B (AKT)/mammalian target of rapamycin (mTOR), signal transducer and activator of transcription 3 (STAT3), and extracellular signal-regulated kinase (ERK) to suppress fibroblast activation and collagen synthesis; downregulating nuclear factor-kappa B (NF-κB) to reduce pro-inflammatory cytokines (IL-6 and TNF-α); activating nuclear factor erythroid 2-related factor 2/heme oxygenase-1 (Nrf2/HO-1) to scavenge ROS and reverse oxidative stress; and inhibiting angiogenesis by modulating vascular endothelial growth factor (VEGF) and platelet endothelial cell adhesion molecule-1 (PECAM-1, CD31). Additionally, they promote fibroblast apoptosis (B-cell lymphoma 2 (Bcl-2)/Bcl-2-associated X protein (Bax) regulation and caspase activation) and reduce α-SMA expression, improving collagen fiber arrangement. Collectively, these anti-fibrotic, anti-inflammatory, antioxidant, and anti-angiogenic effects remold pathological scar tissue.

This study focuses on the latest progress, research hotspots, and future trends of natural medicines in the treatment of pathological scars from January 2008 to July 2025, conducting a bibliometric analysis. By leveraging VOSviewer and CiteSpace software, this study analyzed multiple dimensions of literature in this field, including publication quantity, countries/regions, institutions, journals, authors, references, and keywords, providing valuable insights into the emerging hotspots and trends of research in this domain (Figure 9).

### 4.1 Basic bibliometric information

The research indicates that the annual number of publications has shown an overall upward trend, peaking in 2024. Although only 7 months of data in 2025 were included, the number of publications has been basically equivalent to that in 2024, suggesting that the increasing trend will certainly continue in 2025. The number of citations has exhibited a similar trend to the number of publications, implying that research in this field will remain a major hotspot in the future.

Natural medicines for the treatment of pathological scars have been studied in 39 countries and regions worldwide. The top three active countries/regions in terms of research output are China, the USA, and Iran. China accounts for 58.1% of the total publications, far exceeding other countries. Similarly, 4 of the top 5 authors in terms of publication quantity are from China, and all the top 10 institutions with the highest publication output are from China. This may be attributed to the fact that China is the birthplace of herbal medicine research and the high attention paid by the Chinese government to this field (20). Poland has an average citation count of 65.8, which is much higher than that of other countries/regions, indicating that the overall quality and influence of its published papers are high. Despite China’s leading publication output, its ACs remain relatively low. This may be attributed to limited international collaboration, which restricts the global dissemination of its research findings, in addition to the fact that the majority of its publications are indexed in JCR Q3/Q4 journals with relatively low IF—both of which contribute to the low academic influence and citation rates of its relevant research. While intra-country team collaborations are relatively close, inter-team collaborations remain limited. Therefore, it is advocated to strengthen collaborations between different teams and countries, promote academic exchanges, and break down knowledge barriers.

### 4.2 Research hotspots and Frontiers

Based on the analysis of co-cited references and keyword results, the core research focuses on this field are mainly concentrated in three areas: the treatment of pathological scars using natural medicines, the drug delivery technologies of natural medicines, and the pathological mechanisms of pathological scars (Figure 9).

#### 4.2.1 Common natural medicines in pathological scar treatment

##### 4.2.1.1 *Centella asiatica* and its derivatives

*Centella asiatica* and its derivatives have long been a research hotspot in the treatment of pathological scars using natural medicines, with 17 articles related to them included in this study. *Centella asiatica* contains four main active components: asiaticoside, madecassoside, asiatic acid, and madecassic acid. Madecassoside is a triterpenoid saponin. Investigations have shown that it exerts an inhibitory effect on the proliferation of keloid fibroblasts (KFs) in a way that relies on time and concentration. To induce KF apoptosis through the mitochondrial-dependent pathway, it regulates the expression of Bcl-2 family members, induces mitochondrial membrane potential depolarization, and activates the caspases caspase-9 and caspase-3 (29). Additionally, other studies have indicated that madecassoside can significantly inhibit the migration ability of KFs by suppressing the activity of the p38 mitogen-activated protein kinase (MAPK) and PI3K/Akt signaling pathways, reducing the phosphorylation level of cofilin, and decreasing the formation of F-actin filaments. This suggests its important application potential in the treatment and prevention of HS and keloid (30).

Beyond basic research, Jenwitheesuk et al. (31) conducted a prospective, randomized, controlled, double-blind trial using a *Centella asiatica* extract cream. The study included 30 patients who underwent split-thickness skin graft (STSG) surgery. Patients were administered either a placebo or a cream containing 7% *Centella asiatica* extract at their patients’ donor sites. The Vancouver Scar Scale (VSS) was used for evaluation at 4, 8, and 12 weeks postoperatively, and 23 patients completed the trial. The results showed that the *Centella asiatica* cream group had significant improvements in pigmentation indices and total VSS scores from 4 to 12 weeks. This indicates that it may improve scar pigmentation at STSG donor sites and could be an alternative product for improving HS, although more objective measurements and long-term follow-up are needed for verification.

##### 4.2.1.2 Flavonoids

###### 4.2.1.2.1 Epigallocatechin-3-gallate

EGCG, widely present in green tea, exhibits anti-inflammatory, antioxidant, and anti-angiogenic properties. Five relevant articles were included in this study, with the earliest published in 2008. Through *in vitro* cell experiments and *in vivo* scar models, Park et al. found that EGCG can more effectively reduce KF proliferation, migration, and collagen generation. Its core regulatory mechanism lies in inhibiting the signal transducer and activator of transcription 3 (STAT3) signaling pathway (downregulating the phosphorylation of Tyr705 and Ser727 sites). Although the PI3K and ERK pathways are also inhibited by EGCG, STAT3 is the key regulator of collagen synthesis and cell behavior. *In vivo* experiments further confirmed that EGCG can reduce scar growth and collagen accumulation without obvious cytotoxicity. Klass et al. (19) focused on the effect of EGCG on TGF-β1-mediated wound contraction. Using a fibroblast collagen gel model, they found that EGCG can significantly inhibit TGF-β1-induced collagen gel contraction. It can downregulate the expression of *α*-SMA, reduce the gene expression of connective tissue growth factor (CTGF), regulate the activity of matrix metalloproteinases 1 and 2, and significantly downregulate the gene expression of type I collagen. This indicates that EGCG can regulate collagen metabolism during wound healing through multiple pathways. In addition to basic experiments, Ud-Din et al. (32) conducted a double-blind, randomized controlled trial and innovatively proposed two application methods: “regional pretreatment” (immediate application after wounding) and “direct application” (application after scar formation). The results showed that EGCG can reduce mast cells, inhibit angiogenesis, and increase anti-inflammatory M2 macrophages. It can also improve scar thickness, elasticity, and skin hydration. RNA sequencing further verified that it downregulates inflammation-related factors (interleukin (IL) 17F, IL1A) and angiogenesis-related factors (vascular endothelial growth factor A (VEGFA), CD31). This provides evidence-based support for the clinical application of EGCG and confirms that it can play a positive role from early intervention to post-scar formation treatment.

###### 4.2.1.2.2 Quercetin

As a natural bioactive flavonoid abundant in fruits and vegetables, quercetin is notable for its anti-inflammatory, antiviral, and antioxidant properties. This study incorporated 10 articles related to quercetin.

Si et al. (33) found that KFs have stronger resistance to ionizing radiation (IR) than normal fibroblasts, while quercetin can enhance the sensitivity of KFs to IR in a manner dependent on dose. Additionally, quercetin can downregulate hypoxia-inducible factor 1α (HIF-1α) expression through the phosphatidylinositol 3-kinase/protein kinase B (PI3K/Akt) pathway, and such downregulation of HIF-1α can induce apoptosis in IR-treated KFs. This will provide important molecular mechanism support for quercetin-assisted radiotherapy after keloid resection.

##### 4.2.1.3 Onion extract

Multiple studies have shown that onion extract has various pharmacological properties, including anti-cancer and anti-inflammatory effects, and plays an important role in diseases such as scars, cardiovascular diseases, and respiratory diseases. A total of 13 relevant articles were retrieved in this study. Studies have indicated that different concentrations of enoxaparin and onion extract can all significantly inhibit fibroblast proliferation. High concentrations (500 μg/mL enoxaparin and 1,000 μg/mL onion extract) can also induce cell apoptosis, and both can downregulate the expression of β1 integrin at specific concentrations. However, no synergistic effect on proliferation inhibition was observed when they were used in combination (18). Furthermore, Surakunprapha’s et al. (34) conducted a prospective, randomized, double-blind trial. Over 6 months of treatment, participants were divided into two groups: the experimental group received a silicone gel with added onion, *Centella asiatica*, *Aloe vera*, and *Broussonetia papyrifera* extracts, while the control group received a pure silicone gel. Evaluation using the VSS indicated that at 6 months, the total VSS score of the experimental group was significantly lower than that of the control group. Additionally, the vascular distribution score and pigmentation score of the experimental group were significantly improved compared to the initial baseline.

##### 4.2.1.4 Shikonin

Shikonin is one of the main active components in *Lithospermum erythrorhizon*. Studies have shown that shikonin can inhibit TGF-β1-induced collagen synthesis and cell contraction in hypertrophic scar fibroblasts (HSFs) in a manner dependent on dose. Through suppressing the phosphorylation of extracellular signal-regulated kinase (ERK) and Smad2/3, it downregulates the expression of genes related to collagen (e.g., COL1A1 and COL3A1), which verifies that shikonin can regulate the TGF-β1-mediated abnormal functions of HSFs by means of the ERK/Smad pathway (17). In addition, Liu et al. established an HS model on the ears of New Zealand white rabbits. Experiments demonstrated that shikonin treatment could make the arrangement of scar collagen fibers more regular and the distribution of fibroblasts more uniform while significantly reducing the scar hypertrophy index, fibroblast number density, and collagen fiber area density. It also inhibited fibroblast proliferation by downregulating the expression of proliferating cell nuclear antigen (PCNA) and Ki-67 proliferation antigen, promoted fibroblast apoptosis by upregulating Bax and downregulating Bcl-2, and reduced the synthesis and deposition of collagen I /III and *α*-smooth muscle actin (α-SMA). Furthermore, shikonin significantly downregulated the protein expression of toll-like receptor 4 (TLR4) and phosphorylated nuclear factor-kappa B (NF-κB). These findings confirm that shikonin can improve HS in rabbit ears by regulating the TLR4/NF-κB pathway, providing *in vivo* experimental evidence for its clinical application (35).

#### 4.2.2 Emerging drug delivery methods

##### 4.2.2.1 Microneedles

Research on natural medicines for pathological scar treatment has a long history, and numerous natural medicines have been proven to promote wound healing, prevent scar formation, and eliminate scars. However, the skin barrier results in poor transdermal permeability of drugs, making it difficult for them to penetrate deep into tissues to exert therapeutic effects. Microneedles typically consist of micron-scale needle arrays that cause transient damage to the stratum corneum, forming micropore channels to facilitate transdermal drug delivery. In addition to loading drugs, this physical puncture can also break collagen fibers, interfere with mechanical signaling pathways, and remodel scar tissue. Compared with traditional subcutaneous injection, microneedles are painless, allow self-administration, and improve patient compliance. Microneedles exist in multiple types, such as solid microneedles, coated microneedles, hollow microneedles, and dissolving microneedles. Dissolving microneedles have been extensively studied due to their excellent drug-loading capacity, biocompatibility, and degradability.

The application of microneedles in the field of natural medicines and pathological scars has been widely studied since 2021, with 11 relevant articles included in this study. One study loaded shikonin into dissolvable hyaluronic acid (HA) microneedles, which were prepared using a micro-molding method. These microneedles exhibited sufficient mechanical strength for skin penetration and controllable drug loading. *In vitro* experiments showed that they significantly reduced the viability and proliferation rate of HSFs stimulated by TGF-β1 and downregulated the expression of scar-related genes such as TGF-β1, fibroblast activation protein alpha (FAP-α), and COL1A1, exhibiting local therapeutic effects in both two-dimensional (2D) and 3D cell models (26). Another study by Zhan et al. (36) prepared tanshinone IIA self-dissolving microneedles. Verification showed that it had better transdermal penetration and dermal retention capabilities than tanshinone gel. It could inhibit the proliferation and migration of human skin fibroblasts in a manner dependent on dose, downregulate the expression of TGF-β1, and upregulate the expression of Smad7. Researchers are also making continuous efforts to enhance the loading capacity of microneedles. Younas et al. (37) prepared dissolvable microneedles using mucin—a natural amphiphilic polymer—and loaded them with oregano essential oil (OEO) to construct patches for HS treatment. These patches exhibited sufficient skin/scar tissue penetration ability, rapid skin recovery after removal, good OEO loading, rapid dissolution with easy separation from the basement membrane, and excellent biocompatibility. In the *in vivo* model, these patches significantly reduced scar thickness, epidermal thickness index, scar elevation index, the proportion of collagen area, and the number of capillaries in scar tissue. Biochemical tests further confirmed that it inhibited the expression of TGF-*β*1 and hydroxyproline (HYP). These results demonstrate that OEO exerts anti-HS effects through anti-proliferative and antioxidant activities.

##### 4.2.2.2 Nanomaterials

Guo et al. (15) prepared polyurethane/marine polysaccharide composite nanofiber dressings loaded with 20(R)-ginsenoside Rg3 through electrospinning combined with freeze-drying technology. In a rat third-degree burn model, after 21 days of treatment, these dressings significantly accelerated epidermal closure, alleviated inflammation, promoted vascular maturation, and reduced excessive scar formation by regulating the collagen I/III ratio. This study provides a novel multifunctional dressing for deep burn wound repair and scar inhibition. Chen et al.’s (38) designed and prepared *Salvia miltiorrhiza*−*Blumea balsamifera* nanoemulsion gel for HS treatment. *In vitro* experiments showed that nanoemulsion gel had good stability, with a 24-h transdermal cumulative penetration 2.5 times that of the physical mixture, and could significantly inhibit the proliferation of HSFs. In the rabbit ear HS model, after 21 days of treatment, the experimental group exhibited flatter and softer scars with color closer to normal skin. It reduced the number of fibroblasts and collagen deposition and decreased the collagen I/III ratio. Immunohistochemical results showed that it downregulated the protein expression of TGF-β₁ and Smad2 and improved microvascular distribution by reducing CD34 expression, providing a novel traditional Chinese medicine nano-drug delivery system for HS prevention and treatment.

Owing to the distinctive properties of nanomaterials, they are frequently combined with microneedles to develop combined therapeutic regimens. A research team led by Wu developed a biomimetic transdermal delivery system for nano-drugs: cyclodextrin metal–organic frameworks cross-linked with diphenyl carbonate—loaded with 26% quercetin—were encapsulated in HSF membranes and then dispersed in dissolvable microneedles fabricated from *Bletilla striata* polysaccharide for localized drug delivery. This delivery system enhanced the therapeutic efficacy against HS by modulating the Wnt/β-catenin signaling pathway (Wnt/β-catenin) and Janus kinase 2 (JAK2)/(STAT3) signaling pathways and reducing the expression of type I and III collagen in scar tissue. Its effectiveness outperformed that of systems lacking HSF functionalization or microneedle support. Additionally, it played a synergistic role, demonstrating greater mechanical strength and better physical stability compared to hyaluronic acid-based microneedles. This study thus offers a promising drug delivery strategy for applications in dermatological therapy and cosmetic science (39).

##### 4.2.2.3 Hydrogels

A research team successfully prepared chitosan-alginate hydrogel dressings loaded with *Hypericum perforatum* callus extract. These dressings possess antibacterial, antioxidant, and anti-inflammatory properties. In a BALB/c mouse full-thickness skin wound model, after 14 days of treatment, the wound closure rate reached 97.18%. The dressings promoted epithelial regeneration, granulation tissue formation, neovascularization, and skin appendage repair, with regular and mature collagen fibers. The detection of *α*-SMA indicated no HS formation, providing a novel natural multifunctional hydrogel dressing for wound repair and scar inhibition (40).

#### 4.2.3 Therapeutic mechanisms

Natural medicines exert effects on pathological scars mainly through anti-fibrosis, promoting cell apoptosis, anti-inflammation, antioxidant activity, and anti-angiogenesis. The main pathological features of pathological scars include abnormal proliferation and apoptosis inhibition of fibroblasts, as well as imbalance of collagen metabolism, leading to excessive extracellular matrix (ECM) deposition. Studies have confirmed that 20(R)-ginsenoside Rg3 can significantly reduce the expression of Ki-67 and PCNA, inhibit cell migration, decrease the expression of collagen I/III, downregulate pro-fibrotic genes such as fibronectin and connective tissue growth factor (CTGF), upregulate anti-fibrotic genes such as TGF-*β*3, and increase the ratios of matrix metalloproteinase (MMP) 2/tissue inhibitor of metalloproteinase (TIMP) 1 and MMP9/TIMP1 by regulating the ERK and TGF-*β*/Smad pathways (16).

Persistent infiltration of inflammatory cells, such as neutrophils, macrophages, and lymphocytes, in scar tissue releases IL-6, IL-1β, and tumor necrosis factor-α (TNF-α), which further stimulate fibroblast proliferation and collagen synthesis, forming an “inflammation-fibrosis” vicious cycle. Studies have shown that glabridin can inhibit the development of inflammation, reduce collagen production, and alleviate scar hyperplasia in rabbit ears by regulating the PI3K/Akt and TGF-β1/Smad pathways (41).

Oxidative stress refers to a pathological state in which excessive reactive oxygen species (ROS) are produced in the body or cells, or the function of the antioxidant system is impaired, leading to ROS accumulation and oxidative damage. Arctigenin exerts antioxidant effects mainly by activating the nuclear factor erythroid 2-related factor 2/heme oxygenase-1 (Nrf2/HO-1) signaling pathway. Specifically, it upregulates the content of the antioxidant glutathione and the activity of superoxide dismutase and downregulates the level of the oxidative product malondialdehyde, thereby reversing oxidative stress and redox imbalance (42).

High expression of pro-angiogenic factors, such as VEGF and platelet-derived growth factor (PDGF), in scar tissue leads to increased microvessel density. Shang et al. (14) were the first to verify that artesunate can regulate the immune microenvironment of scars and inhibit endothelial-mesenchymal transition (EndMT) and angiogenesis, and this effect is achieved by inhibiting the TGF-β/Smad and PI3K/Akt/mammalian target of rapamycin (mTOR) pathways.

### 4.3 Clinical translation challenges

Although existing studies have demonstrated the substantial potential of natural medicines in the treatment of pathological scars, their clinical translation is still hindered by numerous unresolved challenges, limiting their widespread advancement in clinical practice.

First, the available clinical evidence is inadequate, with only 12 relevant articles retrieved from 2008 to 2025. The majority of these are small-sample, single-center studies with short follow-up periods. While no severe adverse events have been reported (the majority of reactions are mild cutaneous irritation), it remains difficult to eliminate individual differences and confounding factors. The long-term safety and efficacy of natural medicines have not been fully explored, which restricts their large-scale clinical translation.

Second, natural medicines usually have complex chemical components and are difficult to extract. The majority of active ingredients show low bioavailability. Although advanced drug delivery technologies such as microneedles and nanomaterials have become research hotspots, their clinical application is still in the preliminary stage and requires further validation. In addition, the in-depth mechanisms of pathological scar formation remain insufficiently elucidated, making it difficult to screen highly specific natural medicines and hindering the optimization of clinical therapeutic regimens.

### 4.4 Study limitations

Although our study conducted a comprehensive, multi-dimensional bibliometric analysis of publications on natural medicines for pathological scar treatment retrieved from the WoSCC and PubMed databases and summarized the current research status and trends, several limitations should be acknowledged. First, the data in this study only included English literature, which may lead to potential data gaps. Second, some high-quality articles may have been excluded from the analysis due to insufficient citations, as their publication time is close to the study period. Additionally, 2025 is not yet over, and only 7 months of publication data were included, which may cause potential biases in the annual article statistics. Nevertheless, this bibliometric analysis conducted an in-depth analysis of the available data, provided valuable insights, and offered guidance for future research.

## 5 Conclusion

This study conducted a systematic bibliometric analysis and visualization of studies on natural medicines for pathological scar treatment. It summarized research hotspots, identified the latest progress, and discussed the development prospects of this field. Undoubtedly, natural medicines have become an important research direction for the prevention and treatment of pathological scars. Future research should conduct in-depth studies on the mechanisms by which natural medicines regulate pathological scars, explore combination therapy of multiple natural medicines, and develop better drug delivery methods. At the same time, *in vivo* and clinical studies should be strengthened to verify the effectiveness of natural medicines in the prevention and treatment of pathological scars, and interdisciplinary approaches should be used to promote active collaboration between different research teams.

## Data Availability

Publicly available datasets were analyzed in this study. This data can be found here: https://pubmed.ncbi.nlm.nih.gov/, https://www.webofknowledge.com/.

## Footnotes

1
pubmed.ncbi.nlm.nih.gov
